# Variable suppression by mycorrhiza of root-lesion nematode *Pratylenchus thornei* reproduction among mung bean genotypes has implications for phenotyping

**DOI:** 10.1007/s00572-026-01261-8

**Published:** 2026-04-16

**Authors:** Begita Adhikari, Janak Khadka, Kirsty J. Owen, Elaine C. Gough, Rebecca S. Zwart

**Affiliations:** 1 School of Science, Engineering and Digital Technologies, Centre for Crop Health, University of Southern Queensland, Toowoomba, Queensland Australia; 2 Department of Primary Industries and Regional Development, Perth, Western Australia Australia

**Keywords:** *Pratylenchus thornei*, *Funneliformis mosseae*, Mung bean, Black gram, Arbuscular mycorrhizal fungi, Plant-microbe interactions

## Abstract

Mung bean (*Vigna radiata* and *Vigna mungo*) is susceptible to the endoparasitic root-lesion nematode *Pratylenchus thornei* (*Pt*), which limits crop productivity. Mung bean roots host symbiotic arbuscular mycorrhizal fungi (AMF), which improve nutrient uptake and plant growth. The interaction of these organisms was previously reported in a single mung bean cultivar to increase *P. thornei* reproduction, suggesting potential consequences for phenotyping. Therefore, twelve diverse mung bean genotypes were evaluated to investigate the effect of AMF on *P. thornei* reproduction. A factorial design of genotypes, *P. thornei* and the AMF species *Funneliformis mosseae* (*Fm*) was evaluated in two glasshouse experiments. At 16 weeks after sowing, in mung bean inoculated with *F. mosseae*, there was a significant (*P <* 0.05) decrease in *P. thornei* population densities in nine genotypes whereas no significant effect on *P. thornei* population densities was observed in the remaining three genotypes. Root colonisation by *F. mosseae* was not affected by the presence or absence of *P. thornei*, with consistent root colonisation of ~ 26% across all genotypes. The widest range of *P. thornei* reproduction between genotypes, and hence the best discrimination of responses, occurred without *F. mosseae* inoculation. Consequently, it is recommended that mung bean germplasm should be screened for *P. thornei* resistance in the glasshouse in the absence of AMF for maximal differentiation between genotypes.

**Supplementary Information:**

The online version contains supplementary material available at 10.1007/s00572-026-01261-8.

## Introduction

Mung bean is an important legume crop grown across Asia, Africa, Australia and South America (Nair and Schreinemachers 2020). In Australia, mung bean is mainly grown in Queensland and northern New South Wales (referred to as the northern grains region), with 95% of the 90,000 t of grain produced exported; the industry was worth US$98 million in 2022 (Coriolis Research 2018; Department of Agriculture, Fisheries and Forestry 2023). In Australia, the term mung bean is used to describe both green grams (*Vigna radiata*) and black grams (*Vigna mungo*) (Lawn and Russell 1978).

The yields of many cereal and pulse crops in the Australian northern grains region, including mung bean, are reduced by the root-lesion nematode *Pratylenchus thornei* (*Pt*) (Thompson et al. 1999; Owen et al. 2021). This migratory endoparasite of global economic importance (Mokrini et al. 2019) occurs in 67% of fields in the region (Thompson et al. 2010) and damages root cortical tissues, reducing root function and thereby limiting water and nutrient uptake (Castillo and Vovlas 2007; Thompson et al. 2012; Whish et al. 2014).

The susceptibility of mung bean to *P. thornei* increases nematode population densities during crop growth, with residual populations persisting during fallows and reducing yields of subsequent intolerant crops (Owen et al. 2022). In the northern grains region of Australia, mung bean is often preceded by a winter-grown cereal, such as wheat (Chauhan et al. 2018). *P. thornei* can cause grain yield loss of up to 65% in intolerant wheat cultivars, and 25% in chickpea (Thompson et al. 2012; Reen et al. 2014; Owen et al. 2021). From the perspective of sustainable and profitable farming systems, there is a strong economic imperative to improve resistance in all *P. thornei*-susceptible crops, particularly mung bean given its global importance.

Resistance or susceptibility is characterised by a change in nematode population densities (Starr et al. 2002) and can be assessed efficiently in glasshouse conditions that are optimal for plant growth and nematode reproduction (Thompson et al. 2020). In this way, the genetic potential of the host plant to either restrict or permit nematode multiplication can be measured without limitations. While resistance to *P. thornei* is a quantitative measurement, well-characterised wheat reference genotypes categorised into nine classes from resistant to very susceptible (Thompson et al. 2020) have been used in pulse phenotyping experiments to provide benchmarks for resistance categories (Reen et al. 2019).

The productivity of many crops within farming systems is enhanced by arbuscular mycorrhizal fungi (AMF), which are globally distributed soil-dwelling fungi that develop symbiotic relationships with plants (Thompson 1994; Kivlin et al. 2011; Diagne et al. 2020). Extraradical AMF mycelium extend further into the soil than the root hairs of the host plant and can enhance the plant’s capacity to absorb water, and nutrients, particularly phosphorus (P) and zinc (Zn) (Bhantana et al. 2021). The AMF species *Funneliformis mosseae* (*Fm*) is frequently detected globally, including the northern grains region of Australian farming systems (Jansa et al. 2014; Frew et al. 2025). Natural infection levels of mung bean with AMF communities, dominated by *F. mosseae* in this region have been reported to be up to 98.4% root length colonised (Thompson and Wildermuth 1989). *F. mosseae* belongs to the order Glomerales, and family *Glomeraceae* (Silva et al. 2024), which are known as rapid root colonisers (Oehl et al. 2003).

AMF are known to act as biocontrol agents and reduce the crop damage by plant pathogens, including fungi, bacteria and nematodes (Weng et al. 2022). AMF can induce systemic resistance in host plants, by priming the jasmonic acid (JA) and salicylic acid (SA) pathways, initiating biochemical changes, such as increased production of phenolic compounds, that can prevent nematode penetration (Jung et al. 2012; Desmedt et al. 2020). Furthermore, species within the *Glomeraceae* family typically produce more abundant intraradical hyphae, which could contribute to increased resistance against plant-parasitic nematodes, potentially due to competition for space and nutrients within the root system (Hart and Reader 2002).

AMF may increase, decrease, or not impact *Pratylenchus* spp. reproduction depending on the species and order of the fungus and host plant (Gough et al. 2020). More specifically, species from the genera *Glomus* and *Funneliformis* have been reported to either reduce or have no effect on *Pratylenchus* population densities in host roots (Guillemin et al. 1994; Elsen et al. 2003; Talavera et al. 2001; Forge et al. 2001).

Intriguingly, glasshouse experiments have demonstrated that colonisation by AMF species *F. mosseae* in mung bean cv. Jade-AU was associated with a 2-fold increase in *P. thornei* population densities and improved host nutritional status (P, Zn, and Cu) (Gough et al. 2021, 2022). Increased *Pratylenchus* spp. population densities in plants colonised by AMF have been attributed to increased root biomass (Vaast et al. 1998) and reduced production of plant defence compounds (Frew et al. 2018). It is unknown if the increase in reproduction of *P. thornei* reported in mung bean cv. Jade-AU colonised with *F. mosseae* occurs in other mung bean genotypes. This knowledge is critical for effective phenotyping methods to evaluate the level of *P. thornei* reproduction of mung bean genotypes. Therefore, the current research aimed to evaluate the effect of AMF *F. mosseae* inoculation on *P. thornei* reproduction in diverse mung bean genotypes. Moreover, the distribution and co-existence of *F. mosseae* and *P. thornei* in mung bean roots makes it important to understand their effects across diverse mung bean genotypes, to better inform cultivar selection.

## Materials and methods

### Biological materials

#### Mung bean genotypes

Eleven *V. radiata* genotypes and one *V. mungo* genotype were assessed. Five genotypes AGG324812, AGG326876, AusTRC324277, M07209 and Moong, represent the four sub-populations of the Australian Mung bean Diversity set identified by Noble et al. (2018). Seven genotypes Berken, Celera II-AU, Crystal, Jade-AU, Onyx-AU, Opal-AU and Satin II are commercially released varieties in Australia. Onyx-AU is the only *V. mungo* commercially grown in Australia.

#### *Bradyrhizobium*

The commercially obtained Group I (CB1015) *Bradyrhizobium* in peat (Nodule N New Edge Microbials Pty, Albury, NSW, Australia) was added to all experimental pots as rhizobial symbiont. The number of colony-forming unit (CFU) of *Bradyrhizobium* was determined using the Miles and Misra drop plate method (Vincent 1970). Prior to inoculation, 3 g of the peat containing *Bradyrhizobium* was diluted in 600 mL of water to provide 3 × 10^7^ CFU/mL. Fresh rhizobia dilutions were prepared on the day of inoculation for each of the two experiments.

#### *Pratylenchus thornei*

The *P. thornei* culture was originally collected near Jondaryan, Queensland, Australia (27° 22’ 04” S, 151° 35’ 27” E) and has been maintained in open pot cultures (OPC) at the Centre for Crop Health, Toowoomba, Queensland, Australia (Thompson et al. 2020). The soil and roots were mixed thoroughly, and roots were chopped into ~ 1 cm pieces. Nematodes were extracted from 150 g soil using a modified Whitehead Tray method at 48 h as described in Gough et al. (2021), a week prior to setting up the experiment. The *P. thornei* suspension in water was stored at 4 °C until required.

#### Arbuscular mycorrhizal fungi

The AMF culture used in the experiments was the *F. mosseae* strain ‘Schmelzer 43’, which was originally isolated from Macalister, Queensland (27 02’ 24’’ S, 151° 04’ 12’’ E) (Thompson 1996) and subsequently maintained in OPC using maize (*Zea mays*) for 18 weeks in the glasshouse. The AMF culture was confirmed molecularly to be *F. mosseae* by extracting DNA from a subsample of soil and roots using the DNeasy PowerSoil kit (Qiagen GmBH, Hilden, Germany) following the supplier’s recommendation, followed by PCR using the AMF specific primers WANDA: 5′- CAG CCG CGG TAA TTC CAG CT -3′ (Dumbrell et al. 2011) and AML2: 5′- GAA CCC AAA CAC TTT GGT TTC C -3′ (Lee et al. 2008). The amplified products were sequenced (Macrogen Inc., South Korea). For each OPC, roots were cut into ~ 1 cm lengths and mixed with soil. The spores in a 25 g-subsample were extracted as described in Gough et al. (2021) using the wet sieving and decanting method (Gerdemann and Nicolson 1963) and then quantified under a compound microscope using a Peters counting slide (Peters 1952; Chalex Corporation, UT, USA). After estimating spore density in the subsample, *F. mosseae* inoculum was prepared by mixing soil and chopped roots, and was stored at 4 °C until required.

### Experimental design

Two glasshouse experiments were conducted at the University of Southern Queensland, Centre for Crop Health, Toowoomba, Queensland, Australia (27° 33’ 38.02” S, 151° 57’ 13.90” E) from November 2023 to February 2024. The air and soil temperatures in the glasshouse were maintained at approximately 20–25 °C. The experiments were sown 1 week apart. Each experiment consisted of a full factorial of three independent variables (i) *F. mosseae* inoculation (2 levels, −*Fm* (0 spores/g soil) and +*Fm* (13 spores/g soil)), (ii) *P. thornei* inoculation (2 levels, −*Pt* (0 *Pt*/g soil), +*Pt* (10 *Pt*/g soil)), and (iii) mung bean genotype (11 *V. radiata* genotypes and one *V. mungo* genotype). Twelve mung bean genotypes were included per inoculation treatment, with four inoculation treatments and six replicates. In total, 288 plants were included in the experiment.

Each experiment was laid out in a randomised split-plot design with six replicate blocks, with independent randomisation applied to each experiment. Each block consisted of four main plots to which the four inoculation treatments (−*Pt−Fm*, −*PtFm*, +*Pt−Fm*, +*Pt+Fm*) were randomly assigned. Each main plot consisted of 12 sub-plots to which the 12 mung bean genotype treatments were randomly assigned.

### Experimental conditions

Each pot (150 mm x 70 mm) contained 330 g (dry weight equivalent) of pasteurised Vertosol (Formartin, Queensland, Australia; 27° 23’ 46” S, 151° 26’ 52” E). First the pots were filled with a base layer of 80% of the soil (264 g) mixed with 1 g Scotts Osmocote^®^ Landscape (21.2% N, 1.9% P, 5.7% K and 30.0 Zn mg/kg). The soil chemical properties were pH 8.68 (1 soil:5 water suspension), 2.8 mg nitrate-N/kg soil and 6.8 mg ammonium-N/kg soil from 2 M KCl extraction, 40 mg P/kg soil by Colwell bicarbonate extraction, 534 mg K/kg soil by ammonium acetate extraction and 2.7 mg Zn/kg soil by DTPA extraction (Eurofins APAL Pty Ltd, Burleigh Heads, Queensland, Australia). The addition of Scotts Osmocote^®^ Landscape potentially increased N, P, K and Zn in soil to 652.0, 97.0, 552.0 and 2.8 mg/kg, respectively.

For the pots with the +*Fm* treatment, the soil base layer was mixed with 66 g soil inoculum containing 4,290 *F. mosseae* spores (equivalent to 13 spores/g soil). For the pots with −*Fm* treatment, the soil base layer was mixed with 66 g of soil inoculum that was twice-autoclaved to kill the *F. mosseae* spores. The soil was autoclaved at 121 °C for 30 min, with soil dried between the two runs in a forced-draught oven at 80 °C for 4 h.

Glasshouse benches were set up for bottom watering as described in Sheedy and Thompson (2009). According to the experimental design, the main plots (inoculation treatments) were placed on separated strips of the wicking medium Bidim^®^ (Geofabrics Australasia, Australia) to prevent contamination between inoculation treatments.

After sowing three seeds in each pot, +*Pt* treatments were inoculated with 10 mL of *P. thornei* suspension at the rate of 3,300 *P. thornei*/pot. All pots in the experiment were inoculated with a 1 mL of suspension of *Bradyrhizobium* onto the seeds. The remaining 20% of soil was then added to all pots.

Two weeks after sowing, plants were thinned to one per pot, leaving the roots in the pot. Predatory mites (*Montdorensis* sp. and *Californicus* sp.; Bugs for Bugs, Toowoomba, Queensland, Australia) were spread over the leaves of the plants at 7 weeks to control plant-feeding mites and Veritas-Opti^®^ (Azoxystrobin 222 g/L + Tebuconazole 370 g/L) at label rates was sprayed at 10 weeks to control powdery mildew *(Erysiphe vignae*). The experimental timeline and sampling workflow are shown in Fig. S1.

### Data collection

Each experiment was harvested at 16 weeks after sowing. Four days prior to harvest, the water supply was disconnected to allow the soil moisture content to fall to ~ 45%, to facilitate soil processing (Reen et al. 2019). Plant height was measured from the soil surface to the tip of the highest fully expanded leaf. Plant growth stage was recorded according to Lancashire et al. (1991). The plant shoot was cut just above the soil level and oven dried for 48 h at 105 °C to obtain plant dry biomass. The seeds from mature pods were threshed by hand to obtain the total seed yield per plant (dry seed weight). The pots containing soil and roots were enclosed in plastic bags and stored at 4 °C until processing.

### *Pratylenchus thornei* quantification

For all pots, a 150 g subsample was quantified for *P. thornei* using a modified Whitehead tray method (Whitehead and Hemming 1965; Sheedy et al. 2015). Soil processing, nematode extraction, and quantification were performed as described by Gough et al. (2021). The total *P. thornei*/kg of oven-dried soil equivalent was calculated (Thompson et al. 2020). Hereafter, *P. thornei* population density is expressed as *Pt*/kg.

### *Funneliformis mosseae* colonisation

All samples were evaluated for colonisation by *F. mosseae*. The soil and roots retained from the *P. thornei* extraction were washed over a 250 μm-aperture sieve to collect all the roots present in the 150 g soil sample. The roots were blotted dry with paper towel, the fresh root weight recorded, and the roots were divided into three equal subsamples. For mycorrhizal colonisation, one subsample was stained using the ink-vinegar method (Vierheilig et al. 1998) and observed under an Olympus DF PLAN 2X-2 dissecting microscope to quantify *F. mosseae* colonisation by the grid-intersect method (Giovannetti and Mosse 1980). Another subsample of the roots was dried in the oven at 105 °C for 48 h to calculate the dry root weight of each plant. The remaining subsample was stored at 4 °C but was not required.

### Data analysis

The *P. thornei* count data and *F. mosseae* root colonisation data were transformed to normalise the distribution prior to analysis (Proctor and Marks 1974; Sheedy and Thompson 2009). *P. thornei*/kg were logarithmically transformed by ln(*Pt*/kg + 1) and the percent of root length colonised by *F. mosseae* were arcsine transformed (Gough et al. 2021).

After confirming homogeneity of variance using Bartlett’s test (*P >* 0.05) for the plant growth parameters – plant height, plant growth stage and dry root weight, and *P. thornei* count and *F. mosseae* colonisation, a combined analysis of the two experiments was performed in Genstat (24th Edition, VSN International) using automatic meta-analysis of the serial trials. The parameters of plant dry biomass and dry seed weight did not meet homogeneity conditions were not combined and analysed as separate experiments.

**Fig. 1:**
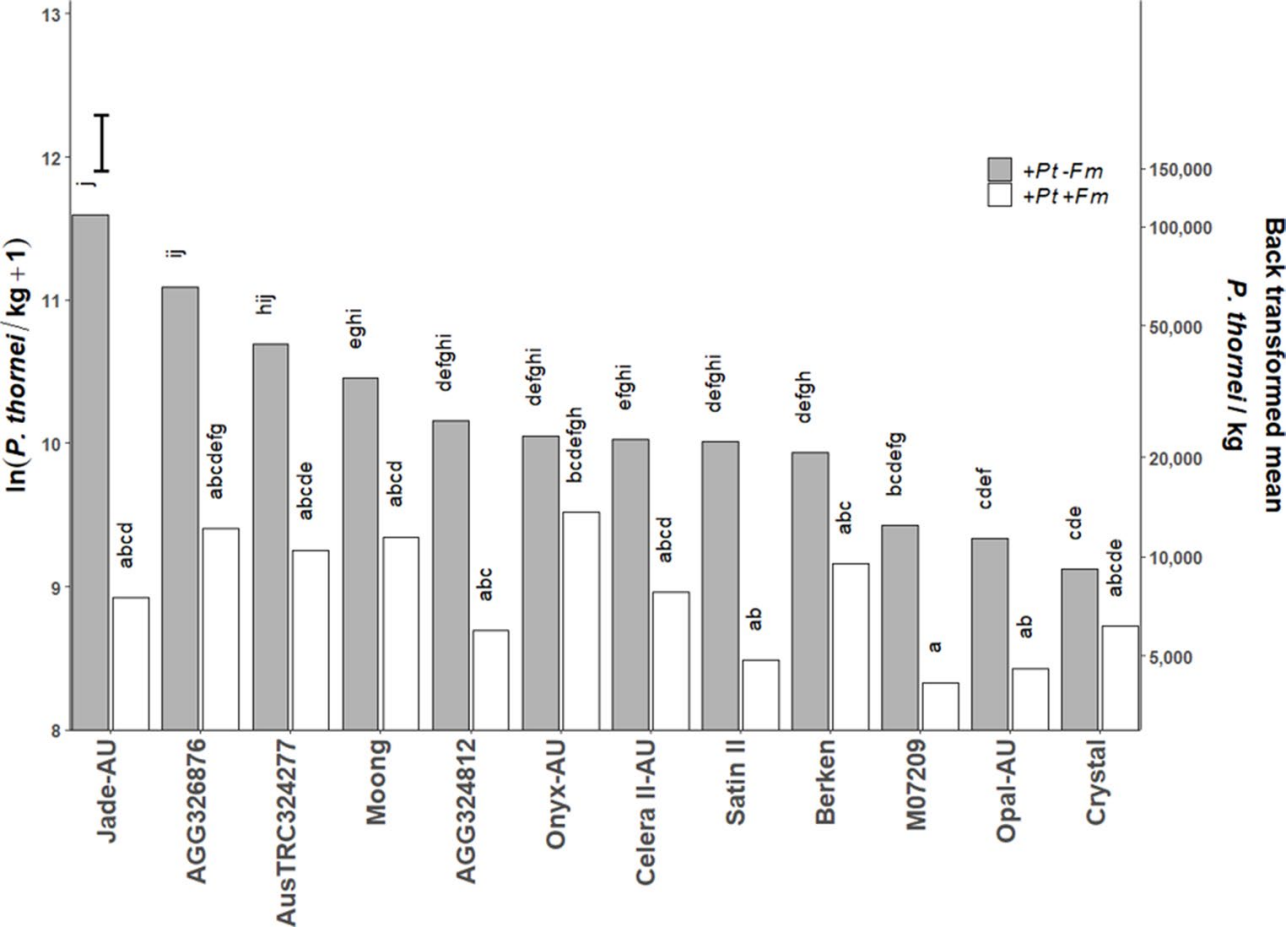
The effect of the interaction of +*Pt-Fm* × +*Pt*+*Fm* × mung bean genotypes on *Pratylenchus thornei* (*Pt*) counts (*P* < 0.001) in a combined analysis of experiments. *Fm* indicates *Funneliformis mosseae*. Grey bars indicate +*Pt*-*Fm*, white bars indicate +*Pt*+*Fm* (*n* = 6). Different letters above each bar indicate significant differences (LSD test, *P* = 0.05) between inoculation treatments and between genotypes. The vertical bar represents the standard error of difference (s.e.d. = 0.39)

The fixed effects in the model included the interaction between inoculation treatment and genotype treatment, while the random effects included blocks nested within inoculation treatment and genotype treatment to account for the split-plot experimental design structure. For significant treatment effects (*P* ≤ 0.05), Fisher’s least significant difference (LSD; *P* = 0.05) test was applied. Following the recommendations of Kozak and Piepho (2020), the standard error of the difference (s.e.d.) from the overall ANOVA is reported as a measure of the precision of treatment comparisons, and s.e.d. bars are presented in Figs. 1, S2 and S3. In addition, inoculation treatments were also analysed as two separate factors (±*Pt* and ±*Fm*) to test main effects of *P. thornei* and *F. mosseae*, as well as their interaction effects with each other and with mung bean genotype. The mean transformed values were back transformed (BTM) for biological interpretation. Regression analyses of *P. thornei* data and each of the plant growth parameters were performed.

## Results

### Plant growth, *P. thornei* and *F. mosseae* parameters

Chi-square statistics and associated probabilities from combined analysis of experiments are presented in Table S1. Treatment means and the probability values from the combined analysis of experiments for main and interaction effects are presented in Table S2 and S3, respectively. Probabilities from combined analysis of experiment with inoculation treatments as two separate factors are presented in Table S4. No contamination of biologicals between treatments occurred. *F. mosseae* colonisation was absent in all −*Fm* inoculation treatments. *P. thornei* were absent in all *−Pt* inoculation treatments.

### Effect of *P. thornei* inoculation and mung bean genotype on *F. mosseae*

For average transformed percent root length colonised by *F. mosseae*, there were no significant differences in the ±*Pt+Fm* inoculation treatment effects (*P* > 0.05), with a mean arcsine transformed value of 0.53 ± 0.17 (BTM = 26% root length colonised) (Table S2). There were also no significant differences among the genotype treatment effects, with average transformed % root length *Fm* colonisation ranging from 0.51 to 0.55 ± 0.013 for Satin II and Jade-AU, respectively (Table S5). No significant interactions effects of inoculation and genotypes were detected (*P* > 0.05). Analysis of inoculations as separate factors also showed that *P. thornei* did not have significant effect on *F. mosseae* colonisation (Table S4) which was consistent with the four-level inoculation treatment analysis.

### Effect of *F. mosseae* inoculation and mung bean genotypes on *P. thornei*

There was a consistent decrease in transformed *P. thornei* counts when plants were inoculated with *F. mosseae* for all mung bean genotypes in the two experiments analysed separately (Figs. S2 and S3), as well as in the combined analysis of experiments (Fig. 1).

For *P. thornei*, there were significant differences between *+Pt* ±*Fm* inoculation treatment main effects (*P* = 0.003), with average transformed value of 10.16 ± 0.86 ( (BTM) = 25,796 Pt/kg) for the +*Pt−Fm* treatment and 8.93 ± 0.86 (BTM = 7,615 *Pt*/kg) for the +*Pt+Fm* treatment (Table S2). There were also significant differences among the genotype treatment effects (*P* < 0.001), with average transformed *P. thornei* counts ranging from 8.91 (BTM = 7,404 *Pt*/kg) for M07209 to 10.29 (BTM = 29,435 *Pt*/kg) for Jade-AU (Table S5). Significant interaction effects between inoculation treatments and genotypes were detected (*P* < 0.001). Analysis of inoculations as separate factors also showed that *F. mosseae* had a significant effect on *P. thornei* (Table S4), which was consistent with the four-level inoculation treatment analysis.

For nine out of the twelve mung bean genotypes the interaction effect resulted in a significant (*P* < 0.05) decrease in *P. thornei* counts in the presence of *F. mosseae*. The magnitude of suppression varied among genotypes. Jade-AU exhibited the strongest response, with a 14.5-fold reduction in *P. thornei* density (108,879 *Pt*/kg in +*Pt*−*Fm* compared with 7,509 *Pt*/kg in +*Pt+Fm*), corresponding to a 93% reduction. AGG326876 showed a 5.4-fold reduction (65,381 vs. 12,172 *Pt*/kg; 81% reduction), and Satin II a 4.6-fold reduction (22,202 vs. 4,855 *Pt*/kg; 78% reduction). The remaining responsive genotypes (AGG324812, AusTRC324277, Moong, M07209, Celera II-AU and Opal-AU) exhibited 2.4- to 4.3-fold reductions in *P. thornei* densities (approximately 58–77% reduction; Fig. 1). In the remaining three genotypes, Berken, Crystal and Onyx-AU, final *P. thornei* counts were numerically higher in the *+Pt−Fm* treatment than in the *+Pt+Fm* treatment, although the difference was not statistically significant.

### Effect of co-inoculation of *P. thornei* and *F. mosseae* on mung bean plant growth

For average plant height, there were significant differences in the four inoculation treatments (*P* = 0.046) and twelve genotype treatments (*P <* 0.001), but no significant interaction effects. The uninoculated plants (−*Pt*−*Fm*) were shorter (mean of 96.30 ± 4.55 cm) than plants inoculated with *+Pt+Fm* (mean of 106.50 ± 4.55 cm). Analysis of inoculations as separate factors showed that *Fm* alone (*P* = 0.007), and *Fm* x Genotype interaction (*P* = 0.020) had significant effects on plant height (Table S4).

For the plant growth stage, there were no significant differences in the four inoculation treatment effects; there was a significant difference in the twelve genotype treatment effects (*P* = 0.006), and no significant interaction effects. The overall mean plant growth stage was 7.90 ± 1.46 with almost all the pods having reached their final stage of development (Table S5).

For dry root weight, there was no significant difference in the four inoculation treatment effects, there was a significant difference in the twelve genotype treatment effects (*P* = 0.045), and no significant interaction effects. The overall mean dry root weight was 4.90 ± 1.42 g.

For dry seed weight and plant dry biomass, there were no significant differences in the four inoculation treatments, nor the twelve genotype treatments and their interaction effects for both Experiment 1 and 2 (data not shown).

### Regression analysis of plant growth parameters with *P. thornei* counts

Regression analysis revealed that there was no correlation between transformed *P. thornei* count and the traits of (i) plant height (y = 0.0098x + 9.1941, *P* = 0.334, R^2^ = 0.093), (ii) plant growth stage (y = 11.99-0.232x, *P* = 0.771, R^2^ = 0.009), (iii) plant dry biomass (y = 0.0408x + 9.637, *P* = 0.342, R^2^ = 0.09), and (iv) dry root weight (y = 0.0514x + 9.8445, *P* = 0.562, R^2^ = 0.035).

## Discussion

This study identified that in a controlled environment, *F. mosseae* colonisation in mung bean reduced *P. thornei* population densities as observed in nine of the twelve genotypes tested. The significant role of *F. mosseae* in reducing the populations of *P. thornei* in multiple mung bean genotypes may be attributed to several interconnected mechanisms. One of the most notable mechanisms is the enhancement of plant health and defence responses (Schouteden et al. 2015). Early colonisation by AMF triggers initial SA associated localised defence in host roots. Once the symbiosis is established, defence signalling shifts towards a JA dominant state, which leads to enhanced phenylpropanoid metabolism, reactive oxygen species modulation and defence related protein accumulation (Jung et al. 2012). Activation of the phenylpropanoid pathway leads to the accumulation of flavonoids and lignin (Wuyts et al. 2006b). Flavonoids have been reported to repel migratory endoparasite *Radopholus similis*, by impairing nematode mobility (Wuyts et al. 2006a). Similarly, increased lignification strengthens root cell walls (Dhakshinamoorthy et al. 2014), enhancing mechanical resistance to penetration and restricting intercellular migration. Because *P. thornei* depends on continuous movement and the breakdown of root cortical tissues, increased phenylpropanoid metabolism may create both chemical and structural barriers, helping to reduce nematode populations in AMF-colonised plants.

In addition, AMF can induce mycorrhiza-induced resistance (MIR), a defence priming effect that enhances protection against pathogens (Cameron et al. 2013). MIR has been shown to reduce infection by *Meloidogyne incognita* in grapevine and tobacco (Cameron et al. 2013), and to enhance resistance in *Musa* spp. against migratory endoparasites such as *R. similis* and *Pratylenchus coffeae* (Elsen et al. 2008). Evidence also suggests that AMF-mediated suppression of nematodes can occur locally at the root level, as demonstrated by reduced infection and reproduction of *Pratylenchus penetrans* in dune grass due to root-level interactions rather than systemic responses (De la Peña et al. 2006).

The lack of *F. mosseae*-mediated reduction of *P. thornei* populations in the three mung bean genotypes Crystal, Berken, and Onyx-Au suggested that the protective effects of AMF are not universal. A systematic review of 60 studies involving wide host range found that the interaction between AMF and *Pratylenchus* spp. varied depending on the crop species, cultivar, and AMF species (Gough et al. 2020). The variability in genotype response in our study aligns with previous studies showing that the efficacy of AMF in suppressing plant-parasitic nematodes is highly dependent on the host genotype (Veresoglou and Rillig 2012). In addition, in the present study, variability of *P. thornei* populations among the mung bean genotypes when inoculated with *F. mosseae* was reduced, resulting in overall a more uniform range of *P. thornei* population densities among genotypes. The AMF mediated mechanisms against biotic stresses described above may impose similar effects on host roots, irrespective of genotypes, thereby leading to homogenisation of *P. thornei* responses across genotypes.

Our results contradict those of Gough et al. (2021; 2022), who reported increased *P. thornei* reproduction in the mung bean genotype Jade-AU co-inoculated with AMF, *F. mosseae*. The decrease in *P. thornei* populations in *F. mosseae*-inoculated mung bean genotypes in our study may be attributed to reduced AMF colonisation, which could be related to soil nutrients, particularly P and Zn. Reduced AMF colonisation, while sufficient to impart defence responses against *P. thornei*, may restrict AMF-induced root growth. This restricted root growth could reduce the availability of feeding and reproduction sites for the nematode, thereby contributing to the observed decline in *P. thornei* populations. Factors such as soil type, and source of AMF and *P. thornei* cultures and experimental conditions were similar to those reported in Gough et al. (2021; 2022). In addition, there is evidence for the effect of nutrients alone including P, Zn and N to decrease *P. thornei* reproduction (Gough et al. 2022; Thompson et al. 2015).

In the present study, the soil had ~ 97 P mg/kg and 2.8 Zn mg/kg (40 mg/kg soil P, 2.71 mg/kg soil Zn plus 1 g Scotts Osmocote^®^ Landscape fertiliser/pot) compared to the study of Gough et al. (2021) in which soil had 45 P mg/kg and 1.45 Zn mg/kg with no additional fertiliser. The total P and Zn were comparatively high in our study due to the addition of fertiliser to ensure non-limiting nutrition for mung bean growth which in turn should also support *P. thornei* reproduction for phenotyping. The levels of P and Zn most likely contributed to the moderate level of *F. mosseae* root colonisation of 26% at 16 weeks after inoculation and contrasts with the relatively high colonisation rate of 60% at 12 weeks after inoculation in Gough et al. (2021). In subsequent research by Gough et al. (2022), AMF colonisation of mung bean cv. Jade-AU decreased by 14% at 11 weeks after the application of 50 mg/kg P applied at sowing, but there was no effect of additional P at 6 weeks and no effect of Zn at either time of assessment. Decreased AMF colonisation with additional P and Zn has also been demonstrated in numerous crop species (Thompson 1987; Saboor et al. 2021; Bate-Weldon et al. 2024; Seymour et al. 2019).The greater levels of P and Zn in our study may have reduced plant dependence on AMF for nutrition, leading to reduced AMF colonisation, consistent with findings that nutrient-limited conditions promote plant dependence on AMF for growth, leading to enhanced root biomass (Wu et al. 2024). In further support of this effect, in our study there was a non-significant effect of *F*. *mosseae* alone on dry root weight.

In our study *P. thornei* did not affect *F. mosseae* colonisation, consistent with findings in mung bean cv. Jade-AU inoculated with *P. thornei* (Gough et al. 2021, 2022) and in *Musa* sp. inoculated with *P. coffeae* (Elsen et al. 2008). However, the effect on *Pratylenchus* spp. differed in these studies, with an increase in *P. thornei* reproduction in plants inoculated with AMF in mung bean (Gough et al. 2022), whereas the *P. coffeae* population was reduced in *Musa* sp. (Elsen et al. 2008), similar to the effect observed in our study.

In our study, and in Gough et al. (2021), *P.* *thornei* and AMF co-inoculation had no significant effect on mung bean dry root weight or plant shoot biomass. However, decreased plant biomass (root and shoot) was reported in a wheat cultivar inoculated with *P. neglectus* and AMF (Frew et al. 2018). Interestingly, a systematic review reported increases in biomass of roots (22 out of 24 studies) and shoots (24 out of 34 studies) in plants co-inoculated with AMF and *Pratylenchus* spp., compared to infection with *Pratylenchus* spp. alone (Gough et al. 2020); no studies showed a decrease in shoot biomass and only two studies reported a decrease in root biomass. These findings demonstrate the complex nature of AMF-nematode-plant interactions, which are influenced by host genotype, AMF strain, nematode species and environmental conditions.

The findings of our study have implications for the standardisation of phenotyping of mung bean genotypes for *P. thornei* resistance. Reliable differentiation between genotypes can be achieved by assessing the true genetic potential for resistance under optimum conditions that maximise *P. thornei* multiplication and plant growth (Thompson et al. 2020). Our study recorded significant differences in *P. thornei* populations within genotypes inoculated with and without *F. mosseae*, where Jade-AU had the most prominent response with a.14.5-fold increase in *P. thornei* populations in the AMF-uninoculated treatment when compared to AMF-inoculated treatment. This magnitude of variation within a genotype highlights how accurate phenotyping is essential to avoid misclassification of genotypes as resistant, particularly in situations where the presence of AMF can suppress *P. thornei* populations and mask genetic resistance. Providing optimum conditions for nematode reproduction, by regulating temperature and the soil environment (Thompson et al. 2015), and maximising plant growth through adequate nutrient and water supply, is essential for consistent phenotyping outcomes (Thompson et al. 2020). While genotypes classified as susceptible in AMF-free screening conditions may not necessarily increase *P. thornei* population densities under field conditions where AMF are present, characterisation of mung bean genotypes, independent of AMF-mediated suppression is essential for identifying the true genetic potential of mung bean genotypes within complex soil biotic environments.

## Conclusion

This study demonstrated that the absence of AMF resulted in significantly higher *P. thornei* populations in nine of twelve mung bean genotypes. Therefore, it is recommended that mung bean germplasm should be screened for *P. thornei* resistance under glasshouse conditions in the absence of AMF to achieve maximum differentiation between resistant and susceptible genotypes when soil P and Zn are non-limiting. There was consistent AMF colonisation across the genotypes irrespective of *P. thornei* inoculation suggesting that the symbiosis between mung bean and *F. mosseae* remains functional even in the presence of *P. thornei.*

## Supplementary information

Below is the link to the electronic supplementary material.

Supplementary Material 1 (DOCX 246 KB)

Supplementary Material 2 (XLSX 31.0 KB)

## Data Availability

All data generated or analysed during this study are included in this published article and its supplementary information files.
