# Supplementary material for: Variable suppression by mycorrhiza of root-lesion nematode Pratylenchus thornei reproduction among mung bean genotypes has implications for phenotyping
Source: Mycorrhiza. 2026 Apr 16;36(2):16. doi: 10.1007/s00572-026-01261-8 (PMC13086660; doi:10.1007/s00572-026-01261-8)
Supplement: Supplementary file 1 — Supplementary Material 1 (DOCX 246 KB) [file 572_2026_1261_MOESM1_ESM.docx]

*Supplementary Figures*
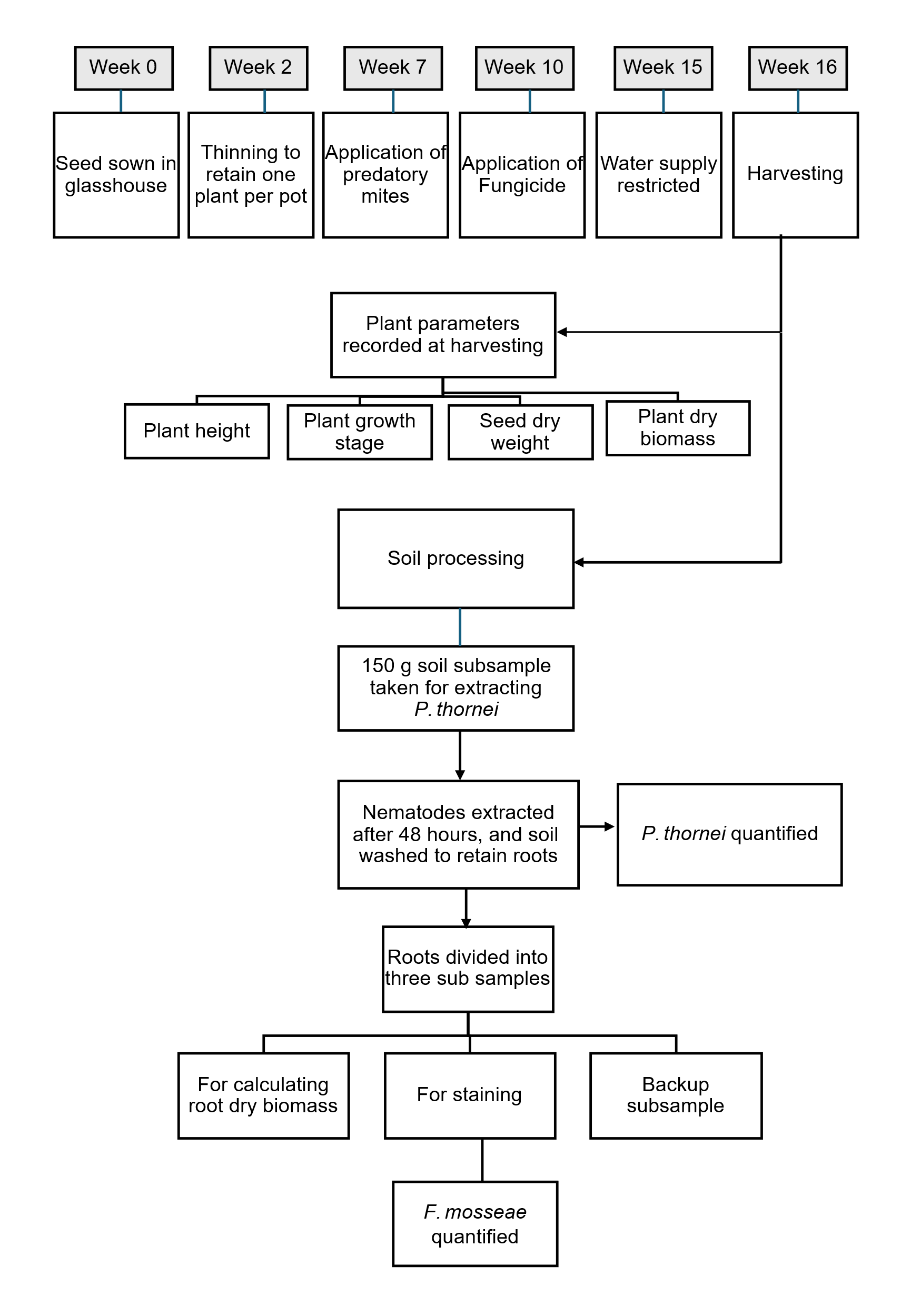


**Fig. S1** Experimental timeline and sampling workflow for assessment of *Pratylenchus thornei* and *Funneliformis mosseae* in mung bean. Plant growth parameters were recorded at harvest (week 16), followed by soil processing for *P. thornei* quantification and root subsampling for dry biomass determination and *F. mosseae* colonisation assessment.


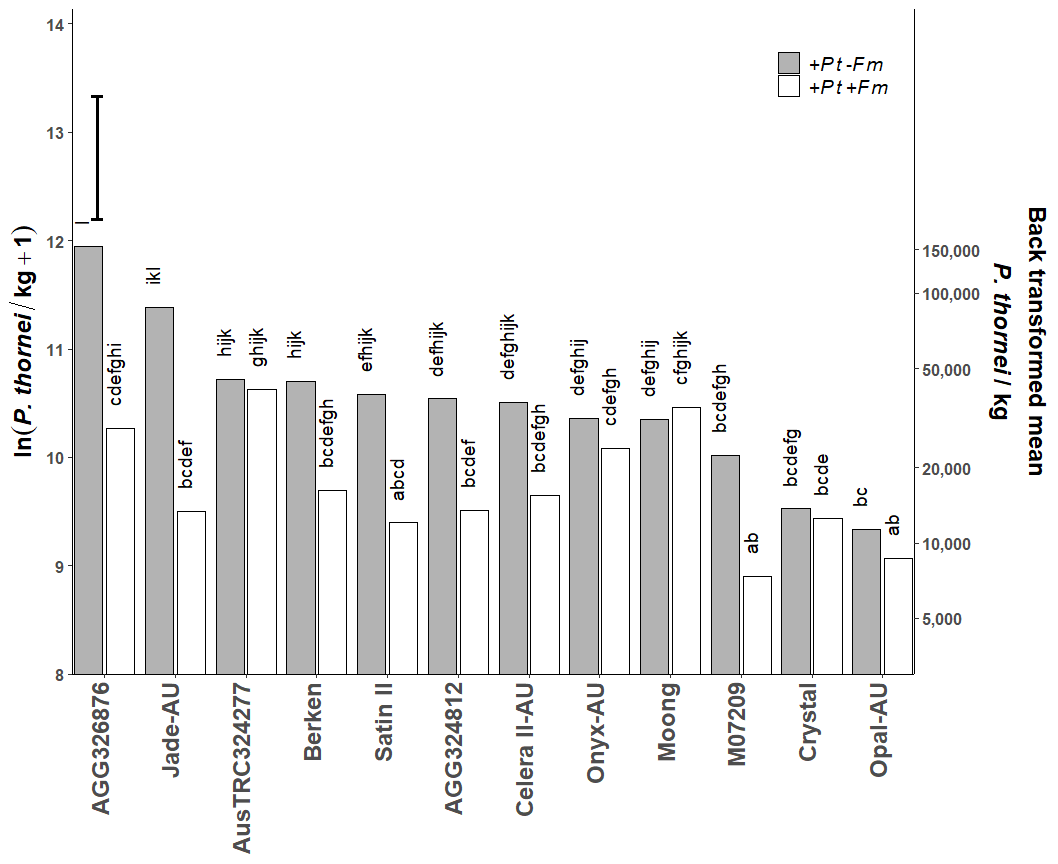


**Fig. S2** The effect of the interaction of *+Pt−Fm* and *+Pt+Fm* inoculation treatments with mung bean genotype on *Pratylenchus thornei* (Pt) counts (P < 0.001) in Experiment 1. *Fm* indicates *Funneliformis mosseae*. Grey bars indicate *+Pt−Fm* and white bars indicate *+Pt+Fm* (*n* = 6). Different letters above bars indicate significant differences between inoculation treatments and between genotypes (LSD test, *P* = 0.05) (sed=0.474).


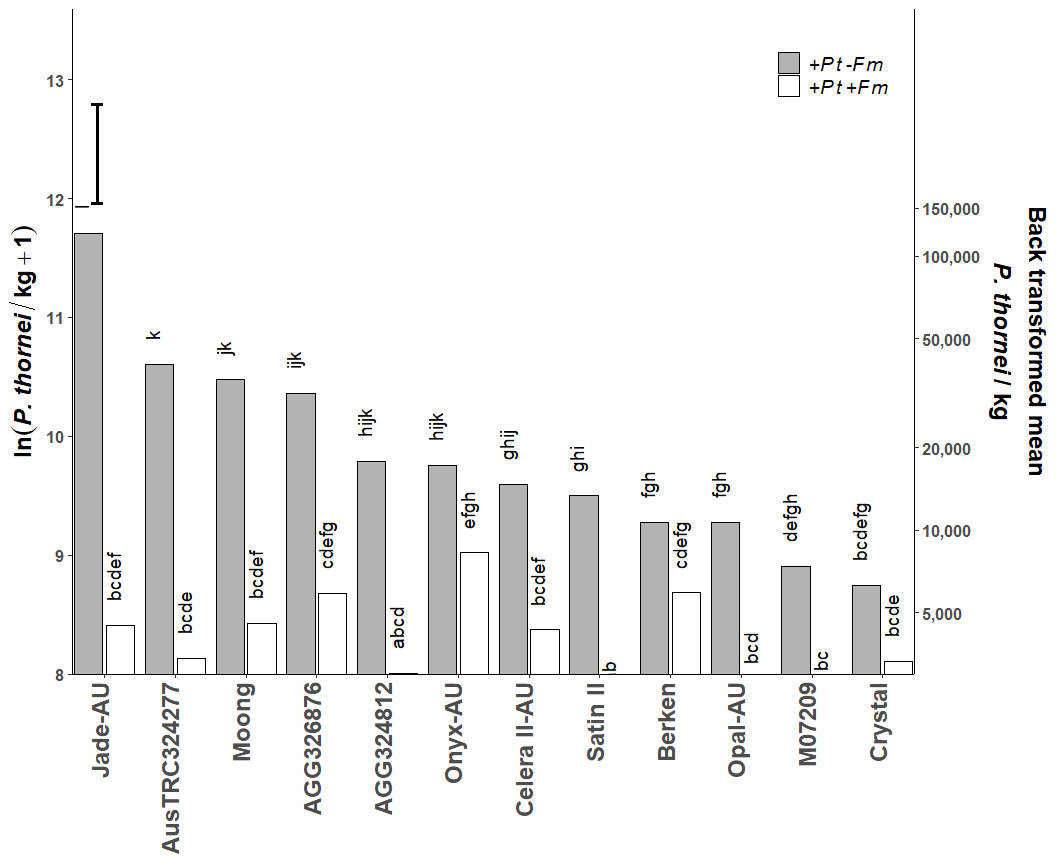


**Fig. S3** The effect of the interaction of *+Pt−Fm* and *+Pt+Fm* inoculation treatments with mung bean genotype on *Pratylenchus thornei* (Pt) counts (P < 0.001) in Experiment 2. *Fm* indicates *Funneliformis mosseae*. Grey bars indicate *+Pt−Fm* and white bars indicate *+Pt+Fm* (*n* = 6). Different letters above bars indicate significant differences between inoculation treatments and between genotypes (LSD test, *P* = 0.05) (sed=0.432).
